# Shaped magnetic field pulses by multi-coil repetitive transcranial magnetic stimulation (rTMS) differentially modulate anterior cingulate cortex responses and pain in volunteers and fibromyalgia patients

**DOI:** 10.1186/1744-8069-9-33

**Published:** 2013-07-02

**Authors:** Alexander Tzabazis, Carina Mari Aparici, Michael C Rowbotham, M Bret Schneider, Amit Etkin, David C Yeomans

**Affiliations:** 1 Department of Anesthesiology, Pain, and Perioperative Medicine, Stanford University School of Medicine, Stanford, CA, USA; 2 Department of Radiology, University of California San Francisco, San Francisco, CA, USA; 3 Sierra-Pacific Mental Illness Research Education and Clinical Center, Veterans Affairs Palo Alto Health Care System, Palo Alto, CA, USA; 4 Cervel Neurotech Inc, Foster City, CA, USA; 5 Department of Psychiatry and Behavioral Sciences, Stanford University School of Medicine, Stanford, CA, USA; 6 Department of Neurosurgery, Stanford University School of Medicine, Stanford, CA, USA; 7 California Pacific Medical Center Research Institute, San Francisco, CA, USA

**Keywords:** Pain, Transcranial magnetic simulation, Acute pain, Chronic pain, Fibromyalgia, Multi coil, Functional imaging, Psychophysics

## Abstract

**Background:**

Repetitive transcranial magnetic stimulation (rTMS) has shown promise in the alleviation of acute and chronic pain by altering the activity of cortical areas involved in pain sensation. However, current single-coil rTMS technology only allows for effects in surface cortical structures. The ability to affect activity in certain deep brain structures may however, allow for a better efficacy, safety, and tolerability. This study used PET imaging to determine whether a novel multi-coil rTMS would allow for preferential targeting of the dorsal anterior cingulate cortex (dACC), an area always activated with pain, and to provide preliminary evidence as to whether this targeted approach would allow for efficacious, safe, and tolerable analgesia both in a volunteer/acute pain model as well as in fibromyalgia chronic pain patients.

**Methods:**

Part 1: Different coil configurations were tested in a placebo-controlled crossover design in volunteers (N = 16). Tonic pain was induced using a capsaicin/thermal pain model and functional brain imaging was performed by means of H_2_^15^O positron emission tomography – computed tomography (PET/CT) scans. Differences in NRS pain ratings between TMS and sham treatment (NRS_TMS_-NRS_placebo_) which were recorded each minute during the 10 minute PET scans. Part 2: 16 fibromyalgia patients were subjected to 20 multi-coil rTMS treatments over 4 weeks and effects on standard pain scales (Brief Pain Inventory, item 5, i.e. average pain NRS over the last 24 hours) were recorded.

**Results:**

A single 30 minute session using one of 3 tested rTMS coil configurations operated at 1 Hz consistently produced robust reduction (mean 70% on NRS scale) in evoked pain in volunteers. In fibromyalgia patients, the 20 rTMS sessions also produced a significant pain inhibition (43% reduction in NRS pain over last 24 hours), but only when operated at 10 Hz. This degree of pain control was maintained for at least 4 weeks after the final session.

**Conclusion:**

Multi-coil rTMS may be a safe and effective treatment option for acute as well as for chronic pain, such as that accompanying fibromyalgia. Further studies are necessary to optimize configurations and settings as well as to elucidate the mechanisms that lead to the long-lasting pain control produced by these treatments.

## Background

Repetitive transcranial magnetic stimulation (rTMS) uses an electromagnetic coil placed over the scalp to induce electrical current pulses within conductive brain tissue in order to modulate the endogenous activity of this tissue. One limitation of current rTMS technology is that deeper brain structures, known to be involved in pain perception, are difficult to target because magnetic field pulse strength rapidly drops as a function of distance from the coil into the brain, and beyond 2–3 cm from the scalp there is insufficient power from a single coil to produce action potentials [1]. Attempts to reach deeper into the brain by increasing power delivered to the coil is not a viable solution, as this approach leads to scalp pain from activation of cutaneous afferents, as well as a relative overstimulation of superficial brain areas, accompanied by risks of off-target effects and seizures. Therefore, most studies trying to utilize rTMS as a treatment for acute or chronic pain have stimulated relatively superficial parts of the brain such as primary motor cortex (M1) [2-6] or the dorsolateral prefrontal cortex [4]. Other groups have used enlarged coil diameters [5-7] in an attempt to reach structures deep within the brain. While enabling the delivery of greater energy levels to depth, this is accomplished at the expense of focality [8] and is thus accompanied by a greater risk of off-target adverse effects [8].

In this study, we used a new approach to specifically target a deeply located brain structure, *i.e.* the dorsal anterior cingulate cortex (dACC), which plays an important role in both cognitive and affective pain perception [9] as demonstrated by fMRI [10] and PET [11] functional imaging studies. In addition to being implicated in acute and chronic pain perception, morphometric magnetic resonance imaging has shown decreases in the dACC in patients with chronic pain conditions, such as fibromyalgia [10,12]. In addition, changes in fractional anisotropy in the dACC as detected by diffusion tensor imaging have been shown with chronic pain conditions as well [13]. Also, real-time fMRI feedback control of the dACC seems to be an evolving method for pain modulation [14].

A novel multi-coil rTMS device (Cervel Neurotech, Foster City, CA) simultaneously activating up to 4 single electromagnetic coils was used to tailor magnetic field pulses towards having the maximal effect on the dACC. We tested the effects of three different coil rotational configurations on acute pain in healthy volunteers using an acute, 10 minute duration thermal pain/capsaicin sensitization model as well as on chronic pain and depression in fibromyalgia patients. Finite element analysis simulations of PET data recorded immediately after each treatment in volunteers was also conducted so as to gain insight into the spatial distribution of the magnitude and direction of energy delivered by each treatment at specific coil power levels used in each individual subject. The coil configuration found to produce the most consistent analgesia in this volunteer study was then used to determine whether similar magnetic stimulation parameters would alleviate chronic pain in fibromyalgia patients.

## Results

### Pain assessment in volunteers

**Figure 1:**
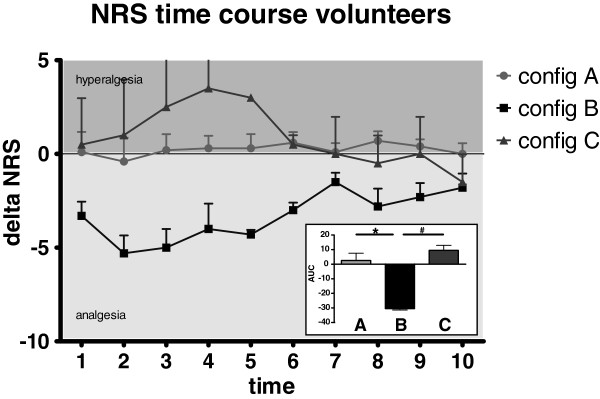
**Time course of numerical pain ratings (rTMS-sham) for each configuration.** The inset shows the averaged area under the curve (AUC ± SEM). *, ^#^: p < 0.05 for configuration B vs. configuration A and configuration C, respectively (one-way ANOVA).

Differences in pain ratings after real and placebo treatment while in the PET scanner are shown in Figure 1 Configuration B produced a significant, robust analgesic effect (AUC −30.5 ± 1.7). For configuration A no clear analgesic or hyperalgesic effect could be observed (AUC 2.6 ± 15.7). Mean area under the curve was significantly lower with configuration B compared configurations A and C indicating a significant analgesic effect for configuration B. Interestingly, configuration C appeared to produce a moderate hyperalgesic effect (AUC 9.5 ± 4.9), potentially indicating an enhancement of pro-algesic structures.

**Figure 2:**
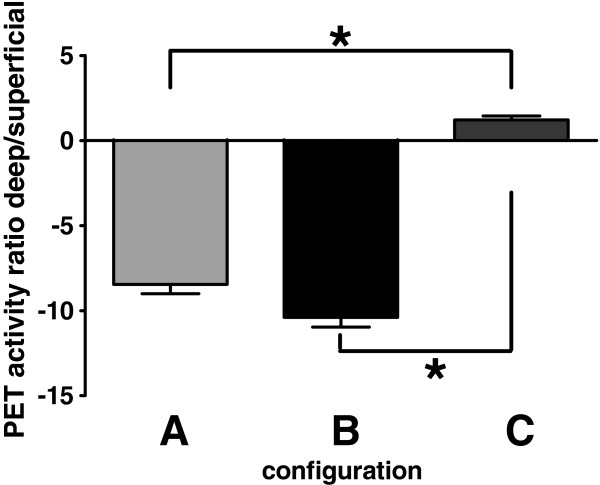
**Ratio of deep vs. superficial PET ROI activity changes (real treatment – sham treatment).** Configurations A and B lead to a marked decrease, whereas configuration C lead to a small increase in activity ratio. *: indicated p < 0.05 (one way ANOVA).

When calculating the activity ratios for average PET activity in the deep (posterior dorsal ACC, anterior dorsal ACC, and the pregenual ACC) versus superficial (supplementary motor area (SMA), preSMA, dorsomedial PFC, and rostromedial PFC) regions of interest in each volunteer, configuration B lead to the biggest decrease in PET activity ratio changes (Figure 2). Consistent with this finding is the observation of the significant analgesic effect after real multi-coil rTMS with configuration B. Interestingly, these changes in PET activity ratio were mostly explained by the almost absent change in activation in the superficial brain areas, -0.04 and 0.125 for configuration A and B, respectively. This indicates that the multi-coil rTMS causes its effect by modulating deep rather than superficial brain structures. Correlating the PET activity ratio with the averaged change in NRS pain ratings during the first 2 minutes (the time period over which H_2_^15^O PET is most sensitive due to the rapid decay) yielded a significant correlation coefficient of 0.61.

### Pain assessment in fibromyalgia patients

**Figure 3:**
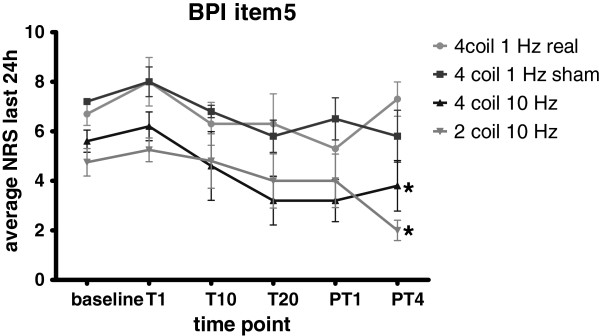
**Time course of BPI item 5 ratings (average pain in the last 24 hours) in fibromyalgia patients for the investigated configurations.** T1, T10, T20: day of 1st, 10th, and 20th treatment session, respectively. PT1 and PT4: 72 hours and 4 weeks after the last treatment.*: p < 0.05 vs. 4coil 1 Hz real (2-way ANOVA with *post hoc* Bonferroni analysis).

Analysis of variance indicated that BPI item 5 was significantly affected by configuration (F(3,5) = 14.42, p < 0.0001) and time point (F(3,5) = 3.83, p = 0.004). There was no significant interaction between configuration and time point. The *post hoc* Bonferroni comparisons showed that compared to the 4 coil 1 Hz real TMS configuration both 10 Hz configurations had significant effects on BPI item 5 at PT4 time point - 4 weeks after the final treatment (Figure 3). Both configurations B and E, applied at 10 Hz showed a progressive analgesic effect that developed over the 4 week time course of treatment sessions. In addition, the pain scores for these two configurations remained significantly attenuated 4 weeks after the final treatment session. BPI item 5 ratings 4 weeks after the last treatment were decreased by 31 and 56% for configuration B and E (both operated at 10 Hz), respectively, compared to the pre-treatment baseline ratings. The 4 coil 1 Hz real TMS treatment group did not significantly differ from the 4 coil 1 Hz sham TMS treatment group (15% decrease for both configurations compared to baseline).

For the secondary outcome measures, analysis of variance revealed a significant effect of time point for changes in Fibromyalgia Impact Questionnaire (FIQ, F(3,3) = 3.74, p = 0.017). Configuration and interaction effects were not significant. Two-way analysis of variance of the Beck Depression Inventory data revealed no significant effects for time point, configuration, and interaction, respectively. No significant changes in between groups were detected in the *post hoc* Bonferroni comparisons for both FIQ and BDI-II (Table 1).

**Table 1:** Fibromyalgia impact questionnaire (FIQ) and the beck depression inventory (BDI-II) ratings over the course of and after the treatments (average ± SD)

**FIQ**	**4 coil 1Hz real**	**4 coil 1Hz sham**	**4coil 10 Hz**	**2 coil 10 Hz**
**T1**	**65.8 ± 8.1**	**72.9 ± 12.0**	**68.7 ± 14.9**	**60.7 ± 14.4**
**T10**	**67.6 ± 10.9**	**64.5 ± 14.3**	**47.4 ± 21.4**	**52.0 ± 21.6**
**PT1**	**56.0 ± 15.9**	**54.3 ± 18.5**	**41.8 ± 15.2**	**51.4 ± 21.9**
**PT4**	**65.3 ± 6.2**	**50.1 ± 19.1**	**37.8 ± 15.8**	**41.2 ± 9.3**
**BDI-II**	**4 coil 1Hz real**	**4 coil 1Hz sham**	**4coil 10 Hz**	**2 coil 10 Hz**
**T1**	**10.3 ± 13.1**	**22.2 ± 12.2**	**12.6 ± 7.4**	**18.3 ± 11.3**
**PT1**	**9.7 ± 11.2**	**15.8 ± 10.0**	**8.0 ± 6.3**	**15.0 ± 11.9**
**PT4**	**14.0 ± 13.1**	**10.8 ± 7.6**	**4.6 ± 5.7**	**9.5 ± 6.4**

T1 and T10: timepoint of 1st and 10th treatment, respectively. PT1: 72 hours after last treatment. PT4: 4 weeks after last treatment. Two-way analysis of variance showed that there was a significant effect of time point for FIQ (F(3,3) = 3.74, p = 0.017). There was no significant effect of configuration and no significant effect of interaction between configuration and time point for FIQ. Two-way analysis of variance for the BDI-II data showed no significant effect of configuration, time point, or interaction.

### Adverse events

No serious adverse events were observed in either the volunteer portion nor in the fibromyalgia patients portion of this study. Adverse events with relatively high incidences (more than 10% of patients, sham vs. real) included scalp pain (11 vs. 2%, p = 0.03), headache (78 vs. 75%, ns), lightheadedness (22 vs. 2%, p < 0.001), back pain (11 vs. 8%, ns), neck pain (0 vs. 13%, p < 0.001), otalgia (11 vs. 4%, ns), nausea (11 vs. 19%, ns), hot flashes (22 vs. 0%, p < 0.001), and pruritus (22 vs. 7%, ns). Interestingly, some of these adverse events (lightheadedness, hot flashes, and scalp pain) occurred with the highest incidence rates in the 4 coil 1 Hz sham rTMS group. Neck pain occurred significantly more often in patients that received real rTMS treatment. There was no significant difference in adverse event rates for occipital pressure (0 vs. 2%, ns), ocular discomfort (0 vs 2%, ns), or vomiting (0 vs 2%, ns) between sham and real rTMS treatment.

## Discussion

This study sought to investigate the analgesic effects of using a multi-coil rTMS array to preferentially target a structure located deeply in the brain, namely the dACC. We used an acute pain model in volunteers to perform functional PET imaging and to investigate effects of a single TMS session on capsaicin-induced thermal hyperalgesia and to establish an optimal coil configuration. The second part of the study exposed fibromyalgia patients to this optimal configuration over 20 treatment sessions, assaying the effects of these treatments on these patients’ pain. In addition, this study examined changes in BDI and FIQ in these patients as secondary outcome measures.

### Volunteer study

Configuration B, one of the three investigated configuration in the volunteer part of the study, yielded robust analgesic effects, whereas configuration C was, if anything, hyperalgesia inducing. The effect of configuration A was highly variable across subjects, with some volunteers demonstrating analgesia, some hyperalgesia, some no effect – thus, for this configuration demonstrated no average effect. This observation might be due to the relatively small size of the targeted brain structure compared with the area of brain influenced by multi-coil rTMS and the vicinity of multiple other critical pain processing centers. Future studies are warranted to investigate the effects of distinct settings of multi-coil rTMS on target structures.

The ratios for deep versus superficial activation were large for configurations A and B. Interestingly, although one would expect to obtain relatively high activation results close to the magnetic coils, the large ratios were mostly explained by the almost absent change in activation of the medial prefrontal cortex, the brain region closest to one of the 4 coils that was usually stimulating with the highest power setting (“top” coil). One possible explanation for the relatively small activation change in the mPFC is that because it may not be primarily activated, but in fact deactivated with the nociceptive input used [15] – unlike the dACC – the region may have been at a resting state or even deactivated during the pain stimulus. Thus and it is possible that the 1 Hz rTMS used is more effective in modulating resting/deactivated cortex rather than affecting an activated state.

### Effect of rTMS settings – configuration and stimulation frequency

**Figure 4:**
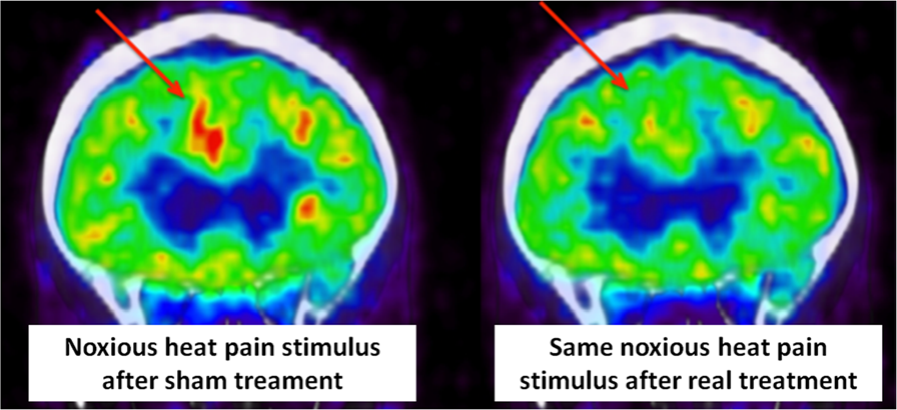
**Three coil configurations used in the PET imaging study are displayed over standard EEG10-20 coil positions.** Large circles represent the coils, and the arrows within represent the direction of the primary electrical current within that coil at the point of contact with the scalp. Note that all configurations have the same four geometric centers for each coil, but the coils are variously rotated so as to shape the magnitude and direction of the resulting magnetic field. The diagram is a planar representation of the curved head surface such that F3, F4 (lateral) and Fpz (anterior) positions are actually approximately vertical and at 90 degrees from the most posterior (“top” coil).

The 1 Hz pulse frequency that showed analgesic effect in the acute pain/volunteer study was not effective for the treatment of chronic pain in fibromyalgia patients. This might be explained by a relatively short-termed effect induced by this stimulation frequency. In volunteers, the acute analgesic effect appeared to last only about 6 minutes on average (Figure 4). The hyperalgesic effect observed after TMS with configuration C showed a similar decrease over time. Those effects are most likely not due to habituation, since similar trends were not observed for configuration A. Another factor that may explain the lack of effect of the 1Hz setting in the fibromyalgia patient population is the difference in age of subjects. In fact, the oldest volunteer was younger than the youngest fibromyalgia patient. Specific configurations might be necessary to obtain desired changes in brain plasticity depending on the age of treated subjects. Future studies should be performed to evaluate effects of age-specific rTMS configurations. A final explanation might be related to morphometric, and presumably cytoarchitectonic structural differences between the dACC of normal volunteers and those of fibromyalgia patients [12]. It may be that the smaller restructured dACC of fibromyalgia patients may be inherently less susceptible to low frequency pulses. Perhaps the most significant finding from these studies is the persistence of analgesic effects for at least 4 weeks after the final treatment session. This effect implies that multiple sessions of rTMS may be inducing neuroplastic processes. It will be critical, in future studies, to determine whether and to what extent these events are recognizable in structural and/or functional scans of similarly treated patients, e.g. reversal of the previously observed morphological differences in the dACC of fibromyalgia patients.

### Extent of analgesic effect

Short et al. [16] tested the effects of left prefrontal cortex rTMS on pain in fibromyalgia patients and observed a significant mean 29% reduction of pain ratings in the real TMS group as compared to a non-significant mean 4% reduction in the sham TMS group. We observed a 31% and 56% reduction of pain ratings for configuration B (10Hz) and configuration E, respectively, at the 4 weeks post treatment compared to 15% for both 4 coil 1 Hz and sham treatment. A possible explanation for this increased analgesic effect compared to the results of Short et al. could be that the shaped magnetic field pulses used in our study were capable of differentially targeting neuronal structures that are more critical in the neuronal pain circuitry (prefrontal cortex vs. dACC) – something that cannot be done using a single coil. However, Short et al. performed only ten rTMS sessions as opposed to 20 sessions in our study and the part of the enhanced analgesic effect could thus also be contributed to the increase in sessions.

For the secondary outcome measures changes in BDI and FIQ, we observed a general trend for FIQ to improve with time, independent of configuration used. It may be that because our patient population started from a relatively low level on both scores, the lack of significance for the BDI-II may be due to a floor effect. It is interesting to note that this general trend of improved FIQ scores seemed to be independent of improvement in pain ratings, i.e. FIQ severity were not directly linked to extent of pain experienced from fibromyalgia. The general trend to improve in the FIQ could also be explained by an increase in patient care while enrolled in the study. An important implication of these results is that, since there was no correlation between improvement in depression and analgesia, it is highly unlikely that the analgesic effects observed were secondary to a treatment-induced anti-depressive effect.

### Appropriate sham rTMS

An inherent problem of rTMS is choosing an ideal sham intervention. Simply placing the magnetic coil over a different brain area or at a different angle might still lead to cortical activation or inhibition and could thus have an effect on the observed outcome parameters [17]. Presenting the characteristic clicking sounds from a recorded audio file as also used in our study may not be an ideal sham procedure since there is no tactile scalp stimulation. However, since multiple coil configurations were used in the volunteer study, with only one consistently producing analgesia, the other configuration/stimulation sets might serve as excellent additional controls. In each case, scalp and auditory sensations would be very similar or identical to those sensed with configuration B. Similarly, despite being effective for acute pain in volunteers, the 4 coil 1 Hz rTMS configuration did not produce a significant analgesic effect in the chronic pain population, but still generated similar sensory cues. In addition, it has been suggested that assessment of duration of analgesic effect might be a better way to discriminate between placebo analgesia and a real treatment effect. In a study performed by Price et al. [18] either saline or a local anesthetic was injected into sympathetic ganglia to alleviate pain from Complex Regional Pain Syndrome (CRPS). While there was no significant difference between peak analgesic effects, the duration of analgesic effect was significantly longer after injection of local anesthetics compared to saline. Transferring these findings to our study, a placebo effect 4 weeks after the final rTMS treatment session seems extremely improbable.

### Potential mechanisms

It was not the primary aim of this study to reveal potential mechanisms of neural responses to magnetic brain stimulation, however we would like to summarize some potential mechanisms of transcranial magnetic stimulation. One should keep in mind that although generally maximum magnetic field intensity is considered to be the most important factor, other factors such as induced electrical fields, spatial summation, potential neuroplastic changes, and differences in tissue/state dependent thresholds may play an important role in mediating rTMS effects. Further studies are warranted to understand the distinct roles that these mechanisms may have in order to optimize future rTMS research and treatment protocols.

The results provided here indicate that multi-coil shaped field rTMS directed toward the dACC might provide a safe and effective means of alleviating both acute and chronic pain. Distinct configurations are required to achieve effects depending on type of pain and/or patient age and possible other factors that still need to be determined. The capacity to differentially target specific structures deep within the brain may suggest numerous additional applications for this relatively new but fast evolving treatment approach of rTMS.

## Conclusions

Multi-coil repetitive transcranial magnetic stimulation was successfully used to reliably and safely affect the dorsal anterior cingulate cortex, a deep brain structures that is critical to pain perception, but which cannot be selectively reached with single-coil approaches. Particular configurations and frequencies of field pulses directed toward the dorsal anterior cingulate cortex reduced acute pain in volunteers and chronic pain in fibromyalgia patients. In addition, the effect on fibromyalgia pain was persistent for at least 4 weeks following treatment, indicating the induction of a neuroplastic, and potentially disease modifying, event.

## Methods

### Volunteer study

#### Subjects

After obtaining informed consent, 16 volunteers were enrolled for the first stage of this study. The average age for the volunteers was 24.8 ± 5.4 years (range 18–38), 5 male and 11 female. Exclusion criteria for the volunteer study were: pregnancy, seizure disorder, use of medications that have the potential of substantially lowering seizure threshold, cardiac pacemaker, electrically conductive implants in the brain, increased intracerebral pressure, minority age, allergy to capsaicin, claustrophobia, actively manifest Axis I psychiatric disorder as assessed though the MINI neuropsychiatric interview by a board-certified psychiatrist, inability to give informed consent, use of analgesics within the previous 2 weeks, acute or chronic pain, and previous experience with TMS. The study enrolling volunteers was approved by the Committee on Human Research, University of California, San Francisco, where the study was conducted.

#### Pain induction and assessment in volunteers

On the scanning day, baseline warmth sensation threshold, heat-pain threshold (HPT_pre_) and maximum heat tolerance temperature (HTT_pre_) were determined for each volunteer using a Peltier thermode (Medoc Ltd, Israel). In order to train volunteers in rating their pain with an 11-point numerical rating scale (0 to 10, NRS), the temperature of the thermode was then set to random temperatures for 5 s between the individual HPT_pre_ and HTT_pre_ separated by 30-s intervals, and the volunteers were asked to rate their pain. To allow for a moderate to strong pain over a 10 minute period while being in the PET/CT scanner without producing skin damage, we then sensitized the skin of the forearm to heat with capsaicin cream (Capsaicin-hp 0.075%, Clay-Parks Lab.) covered with an occlusive dressing. After 20 min, the dressing and remaining capsaicin cream were removed using an alcohol swab and subjects were re-tested for pain threshold (HPT_post_) and tolerance (HTT_post_) and asked to rate their pain as described above. For both PET scans after placebo and TMS, the temperature of the thermode was set to HPT_post_ plus 60% of HTT_post_-HPT_post_. During each 10 minute PET scan, tonic pain was evoked by strapping the Peltier onto the capsaicin-treated skin and maintaining the thermode at this temperature. To assess for effects of rTMS and sham treatments, volunteers were asked to rate their Peltier-evoked pain once per minute during each PET scan.

### TMS device, procedure and configurations

**Figure 5:**
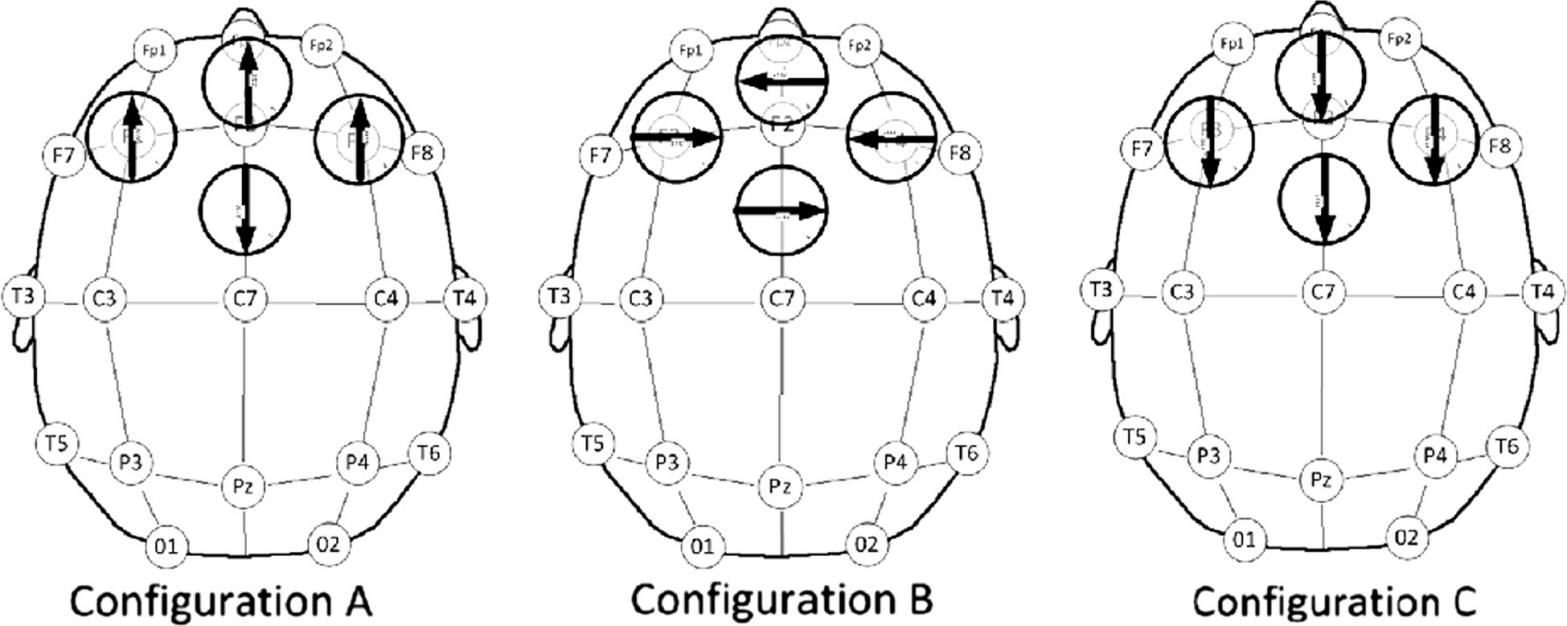
**Representative PET scan images of one subject during noxious heat pain stimulation after sham (left side of the panel) and real (ride side of the panel) multi-coil rTMS treatment.** The red arrow indicates the ACC region. After sham treatment activity in the ACC region seems to be increased by the noxious heat pain stimulus. After real multi-coil rTMS this activation was not observed.

Each subject’s resting motor threshold (MT) was determined by TMS stimulation over the site of the motor cortex, with a positive response in the motor cortex indicated by the minimum power level that produced movement of the subject’s contralateral thumb. To insure subject comfort, maximal TMS power was set to be 110% of motor threshold. However, the subjects’ comfort was assessed throughout the stimulation – if, at any time the patient indicated significant discomfort from the stimulus, the power was decreased incrementally. Simultaneous stimuli produced by pulsed (1 Hz) magnetic fields generated by the four coils produced a composite field the shape of which is dependent on the placement of the four coils on the head as well as the polarity of the fields. The center of each of the coils was at least 4 cm from the center of each other coil, thereby insuring that superficial magnetic fields did not summate above the power of any one coil. The coil configurations tested, A, B and C, shown in Figure 5, were generated using mathematical modeling of the composite field generated by simultaneous activation of 4 coils. Each stimulation session was carried out for a period of 30 minutes and consisted of 1800 pulses.

For the sham procedure, the four coils were placed on the subject’s head in an identical manner to that used in the “real” stimulus session. Thereafter, an audio recording of the characteristic TMS clicking sound was played from speaker mounted behind the top of the subject’s head at a volume comparable with the real treatment and played for 30 minutes.

In order to minimize possible bias, the investigator, who asked the volunteers for the pain rating was not involved in operating the TMS device and the TMS operator was not involved in the volunteers’ pain assessment for studying treatment effects. TMS operator did, however, in each case, ask subjects about possible unpleasantness/scalp pain induced by the TMS treatment.

As TMS produces very loud clicks, all subjects wore ear plugs at all times while receiving real and sham treatments to prevent ear damage.

### H_2_^15^O PET scan

After obtaining intravenous access, subjects were transferred to the scanner (GE Discovery 64 slice PET/CT), which was approximately 1 meter from the rTMS chair. There the subjects laid supine, head first, arms down. After advancing their head into the scanner, subjects were instructed to remain still, while a scout scan or topogram was acquired to determine the field of view (~16.2 cm) for the CT and PET, respectively. After preparation by a near-by cyclotron, a 10 mCi dose of H_2_^15^O in a small volume of normal saline (1–2 ml) was intravenously administered and flushed with 10 ml of normal saline. Immediately after, a single bed image of the brain was acquired for 10 minutes in dynamic mode. H_2_^15^O was produced in the in-house cyclotron installed at the China Basin facilities of UCSF following human administration standards and after RDRC approval. Due to the short half-life (t_1/2_) of ^15^O (122 seconds), a brain PET scan can be performed several times in a single experimental session with the patient being in different pain states (baseline, pain with sham treatment, and pain with real treatment). Data were normalized, and appropriate corrections were applied for dead time, randoms, scatter, and attenuation.

### Volume of interest and finite element analysis

Using computerized finite-element analysis (FEA), software (Opera 3D, Cobham Technical Services, Oxfordshire, England, U.K.) simulations were conducted for each specific coil configuration, and the power levels received by each individual subject in the acute and FMS pain studies. The peak magnetic field spatial profile was then computed for a series of volumes of interest in the brain that was correlated with the analgesic effect produced by specific coil configurations. From this matrix, values representing peak magnetic field magnitude and direction were extracted from locations corresponding with the dorsolateral aspect of the prefrontal cortex (dLPFC), the dorsal anterior cingulate cortex (dACC), and the cortical surface. Changing the x, y and z directional profiles illustrated the steerability of the field pulses at depth and the unique magnetic field shape created by each coil configuration. The results were also used to verify safe power settings for individual coils.

### Fibromyalgia patient study

#### Subjects

Sixteen patients were included in the fibromyalgia study. Ages for the patients ranged from 40–64 years (mean 53.2 ± 8.9), 14 female and 2 male. Inclusion criteria were: fibromyalgia diagnosis according to the American College of Rheumatology criteria, moderate to severe pain (defined as *Brief Pain Inventory* – BPI - item 5 rated as 4 or greater) from fibromyalgia despite current and stable treatment regimen, agreement not to become pregnant during the study. Exclusion criteria were seizure disorder, metal implants on or in the brain, spinal cord, ear, eye or heart, current use of potentially proconvulsant medications, medication of oral amitriptyline > 100 mg/day, non-scheduled (PRN) analgesics, anticonvulsant or antidepressant medications, use of opioid analgesics during study participation, severe depression or suicidality, other significant psychiatric disorder, and previous experience with TMS. The fibromyalgia patient study was approved by Shulman Associates IRB (ClinicalTrials.gov identifier: NCT01229852).

#### Assessment in fibromyalgia patients

Fibromyalgia patients were assessed using the *Brief Pain Inventory* (BPI) questionnaire, the *Fibromyalgia Impact Questionnaire* (FIQ) [19] and the *Beck Depression Inventory* (BDI-II) [20].

The primary outcome measure in fibromyalgia patients was change in item 5 of BPI, *i. es* average pain rating in the last 24 hours, at the post-treatment time point 4 (4 weeks after last treatment session).

Secondary outcome measures included changes in FIQ and BDI-II from the baseline.

### TMS procedure and configuration in fibromyalgia patients

Determination of motor threshold and safety precautions were performed as described for the volunteer study.

The “B” coil configuration used with most fibromyalgia patients was that found to be most effective in the volunteer study component – but applied at different either 1 or 10 Hz, rather than all at the1 Hz frequency used in the volunteers. Thus, the following configurations were tested in fibromyalgia patients: 1) configuration B, 1 Hz, 2) sham configuration B, 1 Hz, 3) configuration B, 10Hz. In addition, a 2 coil, 10 Hz configuration designated “E” was designed to mimic the field shape produced by configuration B, but with less specificity.

Patients received 20 daily treatment sessions of 30 minutes each over a 4 week period.

### Statistical analysis

All data are presented as average ± SEM.

For the volunteer study, differences in NRS ratings of volunteers (NRS_real, min1_ – NRS_sham, min1_, etc.) were calculated for each minute while subjects were in the PET scanner. To minimize the problem of multiple comparisons, the area under the curve (AUC) was calculated for each individual subject with the following formula:

$$\mathrm{AUC}=\frac{1}{2}\sum_{i=0}^{n-1}\left(t_{i+1}-t_{i}\right)\left(y_{i}+y_{i+1}\right)$$

where *y*_*i*_ represents the difference in NRS ratings (real TMS – sham TMS) reported at times *t*_*i*_ (i = 0,…,n) [21]. AUCs for pain NRS were then averaged according to configuration and tested with one-way ANOVA with post hoc Bonferroni comparisons for significant differences.

For the imaging data, three deep and four superficial regions of interest (ROI) were defined: (1) deep ROI: posterior dorsal ACC, anterior dorsal ACC, and the pregenual ACC (2) superficial ROI: supplementary motor area (SMA), preSMA, dorsomedial PFC, and rostromedial PFC [22]. Activity changes (rTMS treatment – placebo treatment) were averaged for the deep and superficial ROIs, respectively, and a ratio of deep versus superficial average change was calculated for each of the investigated coil configurations. The Pearson product–moment correlation coefficient was calculated for PET activity change ratios and changes in pain perception as measured by NRS ratings during the first 2 minutes of the H_2_^15^O PET scan accounting for the short half-life of ^15^O.

For the fibromyalgia study, data was analyzed using a repeated measures two-way ANOVA with Bonferroni *post hoc* comparisons for the primary and secondary outcome measures.

Incidences of treatment-related adverse events were tested for significance using chi^2^ test with Yates’ correction. For this analysis, data for all real rTMS groups (4coil 1Hz, 4coil 10Hz, and 2coil 10Hz) was pooled and compared to the 4coil 1Hz sham treatment group.

The significance level for all statistical tests was set to p < 0.05.

## Competing interests

AT has in the past functioned as paid consultant to Cervel Neurotech Inc. and owns stock options.

CMA has no competing interests.

MCR has in the past functioned as paid consultant to Cervel Neurotech Inc.

MBS is founder and Chief Medical Officer, employee of Cervel Neurotech Inc. He owns stock in the company and has intellectual property rights.

AE has no competing interests.

DCY has in the past functioned as paid consultant to Cervel Neurotech Inc. and owns stock.

## Authors’ contributions

AT helped in designing the study, carried out psychophysical testing, analyzed data and drafted the manuscript. CMA carried out PET/CT imaging and helped in drafting the manuscript. MCR helped in designing the study and drafting the manuscript. MBS helped in designing the study, performed TMS treatments, analyzed data and helped in drafting the manuscript. AE analyzed data and helped in drafting the manuscript. DCY designed the study, carried out psychophysical testing, analyzed data and helped in drafting the manuscript. All authors read and approved the final manuscript.
