# Correction: shaped magnetic field pulses by multi-coil repetitive transcranial magnetic stimulation (rTMS) differentially modulate anterior cingulate cortex responses and pain in volunteers and fibromyalgia patients

**DOI:** 10.1186/1744-8069-10-16

**Published:** 2014-03-04

**Authors:** Alexander Tzabazis, Carina M Aparici, Michael C Rowbotham, M B Schneider, Amit Etkin, David C Yeomans

**Affiliations:** 1 Department of Anesthesiology, Pain, and Perioperative Medicine, Stanford University School of Medicine, Stanford, CA, USA; 2 Department of Radiology, University of California San Francisco, San Francisco, CA, USA; 3 Sierra-Pacific Mental Illness Research Education and Clinical Center, Veterans Affairs Palo Alto Health Care System, Palo Alto, CA, USA; 4 Cervel Neurotech Inc, Foster City, CA, USA; 5 Department of Psychiatry and Behavioral Sciences, Stanford University School of Medicine, Stanford, CA, USA; 6 Department of Neurosurgery, Stanford University School of Medicine, Stanford, CA, USA; 7 California Pacific Medical Center Research Institute, San Francisco, CA, USA

## Correction

In our recently published article [1] we made a mistake in describing the contribution of two of the authors. In particular, we indicated that AT helped in designing the study, carried out psychophysical testing, analyzed data and drafted the manuscript and that DCY designed the study, carried out psychophysical testing, analyzed data and helped in drafting the manuscript. This was an inadequate description of the contribution of these two authors. In fact however, neither AT nor DCY actually carried out the psychophysical testing. Rather they trained others in the methods and oversaw the results.

In addition, there were some problems with the published figure legends that were not seen in the proofs. Thus, some of the figures and their legends are misappropriated with regards to Figure 4 (page 5) and Figure 5 (page 7). The legend associated with the image in Figure 4 should be the legend that is now incorrectly associated with Figure 5, and vice-versa. Also, on Page 4 of 9, "Effect of rTMS settings" incorrectly refers to Figure 4. This should refer to Figure 1.
